# Interventions used to reduce infectious aerosol concentrations in hospitals—a review

**DOI:** 10.1016/j.eclinm.2024.102990

**Published:** 2024-12-18

**Authors:** Gráinne Brady, Fiona Bennin, Rosaline De Koning, Cecilia Vindrola-Padros, Sigrún Eyrúnardóttir Clark, Manish K. Tiwari, Simon Watt, Andrea Ducci, Ryo Torii, Danielle Morris, Elizabeth Lloyd-Dehler, Jerry Slann, Fiona Stevenson, Zarnie Khadjesari, Hakim-Moulay Dehbi, Lena Ciric, Ruth Epstein, John Rubin, Catherine F. Houlihan, Rachael Hunter, Laurence B. Lovat

**Affiliations:** a Department of Targeted Intervention, Rapid Research Evaluation and Appraisal Lab (RREAL), University College London, UK; b Department of Mechanical Engineering, University College London, UK; c WEISS Centre, University College London, UK; d East and North Hertfordshire NHS Trust, UK; e Lay Member, UK; f Institute of Occupational Medicine, UK; g Institute of Epidemiology and Health Care, University College London, UK; h School of Health Sciences, University of East Anglia, UK; i Comprehensive Clinical Trials Unit, University College London, UK; j Department of Civil, Environmental and Geomatic Engineering, Healthy Infrastructure Research Group, University College London, UK; k Royal National Throat Nose and Ear Hospital, University College London Hospitals NHS Foundation Trust, London, UK; l Department of Virology, University College London Hospitals NHS Foundation Trust, London, UK; m Division of Infection and Immunity, University College London, UK; n Division of Surgery and Interventional Science, University College London, UK

**Keywords:** Infectious respiratory particles, Hospital, Filtration device, Ventilation device, Ventilation, Air safety

## Abstract

**Background:**

The COVID-19 pandemic highlighted the need for improved infectious aerosol concentrations through interventions that reduce the transmission of airborne infections. The aims of this review were to map the existing literature on interventions used to improve infectious aerosol concentrations in hospitals and understand challenges in their implementation.

**Methods:**

We reviewed peer-reviewed articles identified on three databases, MEDLINE, Web of Science, and the Cochrane Library from inception to July 2024. 6417 articles were identified, 160 were reviewed and 18 were included.

**Findings:**

Results on aerosol concentration were discussed in terms of three categories: (1) filtration and inactivation of aerosol particles; (2) effect of airflow and ventilation on aerosol concentrations; and (3) improvements or reduction in health conditions. The most common device or method that was outlined by researchers was high efficiency particulate air (HEPA) filters which were able to reduce aerosol concentrations under investigation across the included literature. Some articles were able to demonstrate the effectiveness of interventions in terms of improving health outcomes for patients.

**Interpretation:**

The key finding is that infectious aerosol concentration improvement measures based on filtration, inactivation, improved air flow dynamics, and ventilation reduce the likelihood of nosocomial infections. However limitations of such approaches must be considered such as noise pollution and effects on ambient humidity. Whilst these efforts can contribute to improved air quality in hospitals, they should be considered with the other interacting factors such as microclimates, room dimensions and use of chemical products that effect air quality.

**Funding:**

This study is funded by the 10.13039/501100000272National Institute for Health and Care Research (NIHR) (NIHR205439).

**Research in context**

**Evidence before this study**

Ventilation approaches have been recommended to prevent airborne transmission of infectious respiratory particles, however natural ventilation is not always possible in the context of NHS hospitals. The literature was searched in July 2023, and re-run in July 2024, on the databases MEDLINE, Web of Science, and the Cochrane Library, using search terms based on ‘respiratory infection’, ‘filtration’, ‘recirculation’, and ‘airflow’. Publications were included if they focused on interventions within the acute care setting that improved aerosol concentrations and respiratory infections, with no restrictions in language. Risk of bias was assessed using the Mixed Methods Appraisal Tool.

**Added value of this study**

There were 18 studies that were able to demonstrate the impact of using filtration and ventilation interventions, with the majority finding improvements in aerosol concentrations and health conditions. The most frequently referenced intervention was HEPA filters.

**Implications of all the available evidence**

The negative impact of these devices should be studied further such as their impact on noise pollution and on ambient humidity. Future research on improving air quality should recognise there are a host of factors that interact with each other to effect air quality, such as microclimates, ventilation, filtration, use of chemical products, room dimensions, and the number of people and items within a room.

## Introduction

Transmission of severe acute respiratory syndrome coronavirus 2 (SARS-CoV-2) can occur through airborne transmission of infectious respiratory particles (IRPs).^1^ The World Health Organisation (WHO) defines IRPs as infectious particles that travel through the air by expired airflow from an infectious individual through coughing, sneezing, talking, and breathing which then enter another human's respiratory tract.^1^

IRPs can be transmitted through the air through two routes.^1^ The first route is airborne transmission which involves the IRPs travelling short or long distances based on factors such as ventilation, airflow, temperature, and humidity.^1^ Direct deposition is the other route of transmission that involves the IRPs being expelled into the air and then directly deposited onto another person's mouth, nose or eyes.^1^

IRPs that are larger than 100 μm when exhaled most often drop to the ground once they have been expelled by two metres and are therefore difficult to inhale, and instead rely on transmission via fomite exposure (touching an infected surface to a person's eyes or mouth).^2^ IRPs of this size therefore only have a near-field mode of transmission. IRPs that are smaller than 100 μm when expelled typically start to evaporate to decrease in size. These smaller IRPs can be transported by air currents at distances further than two metres before falling to the ground, and therefore have the ability for far-field transmission, in addition to near-field.^2^^,^^3^

During the COVID-19 pandemic many hospitals identified SARS-CoV-2 RNA in the air.^2^ In July 2020, the WHO reported airborne transmission of SARS-CoV-2 as a transmission route, likely happening in healthcare settings and crowded indoor areas.^4^ Approaches such as ventilation in which old potentially contaminated air is exchanged for clean air has been recommended.^5^ However, such recommendations are not always achievable as many hospital rooms especially within the context of the NHS do not have windows that can be opened or mechanical ventilation systems.^5^ To retro-fit such structures would be costly, could impact the ability for an institution to provide round the clock patient care, and may require staff training to implement such systems.^6^, ^7^, ^8^, ^9^

The aim of this review was to map the existing literature on interventions used to reduce aerosol concentrations that contain or simulate respiratory infections in hospitals, as well as to better understand challenges in implementation.

## Methods

The review was designed following the approach for rapid evidence reviews^10^ with scope to incorporate relevant grey literature. The review followed a phased approach, which begins with a broad search strategy that is expanded with each round of searches. We followed the Preferred Reporting Items for Systematic Reviews and Meta-Analysis (PRISMA) statement to guide the review design and the reporting of the methods and findings. No protocol was published; however, an internal project proposal was developed.

The research questions guiding the review were:

RQ1: What are the types of interventions currently being used to improve aerosol concentrations in hospitals?
RQ2: Have any of these been evaluated? If so, what are the main evaluation findings?
RQ3: What are the main lessons learnt from the implementation of these interventions?

### Search strategy and selection criteria

The search strategy was developed by researchers and relevant clinical colleagues. The first phase of the search strategy was broad and was run on general databases such as Google Scholar and PubMed (Appendix S1). This led to the selection of a preliminary list of resources. This list was scanned for relevant key terms. The final search strategy is available in Appendix S2.

The search was not limited in any way other than to streamline outcomes, interventions, and the environment of the study. There was no date, language or location limitation. Final searches were conducted at the end of July 2023, the search was re-run at the end of July 2024 (to capture any published data between that period) on three databases (MEDLINE, Web of Science, and The Cochrane Library).

The search results were imported into EndNote and duplicates were removed. Once this was complete, all included references were imported into Rayyan for screening.

Four researchers conducted the title and abstract screening process by each screening a proportion of the publications. The researchers then cross-checked 10% of each other's exclusions against the inclusion criteria (available in Appendix S3). The remaining publications that met the inclusion criteria were organised and allocated between the researchers to facilitate full text screening. The same process was followed where each researcher screened a portion of the sample and then cross-checked 10% of each other's exclusions.

The following inclusion criteria were applied:

Peer reviewed articles where interventions improving aerosol concentrations are mentioned in the context of hospital settings, this could also be discussed in terms of a respiratory virus/infection.
No restriction on date, language, or study location.
To ensure the search was manageable, we did not include articles related to aerosol concentration in any other environment, PhD theses, dissertations, books, conference proceedings, incomplete versions, articles where we could not access the full text or letters to editors. The full inclusion criteria is available in Appendix S3.

### Data extraction and synthesis

Data extraction was conducted using Microsoft Excel to organise the review process. Data was extracted by four reviewers who each extracted data from a portion of the publications, the extraction form was piloted with two initial studies, and amendments were made before extracting data from all included studies. All the data extraction was cross-checked by another reviewer. Data were synthesised using narrative synthesis.^11^

### Quality assessment

The methodological quality of the empirical articles was critically appraised using the Mixed Methods Appraisal Tool (MMAT).^12^ Again, four researchers quality appraised a portion of the publications each, and appraisal between researchers was cross-checked. The MMAT was developed to allow systematic reviewers to assess the methodological quality of diverse study designs, including qualitative, quantitative, and mixed methods.

### Role of funding source

The study's funder was not involved in the design, data collection, analysis, interpretation, or manuscript writing.

## Results

### Article selection

**Fig. 1:**
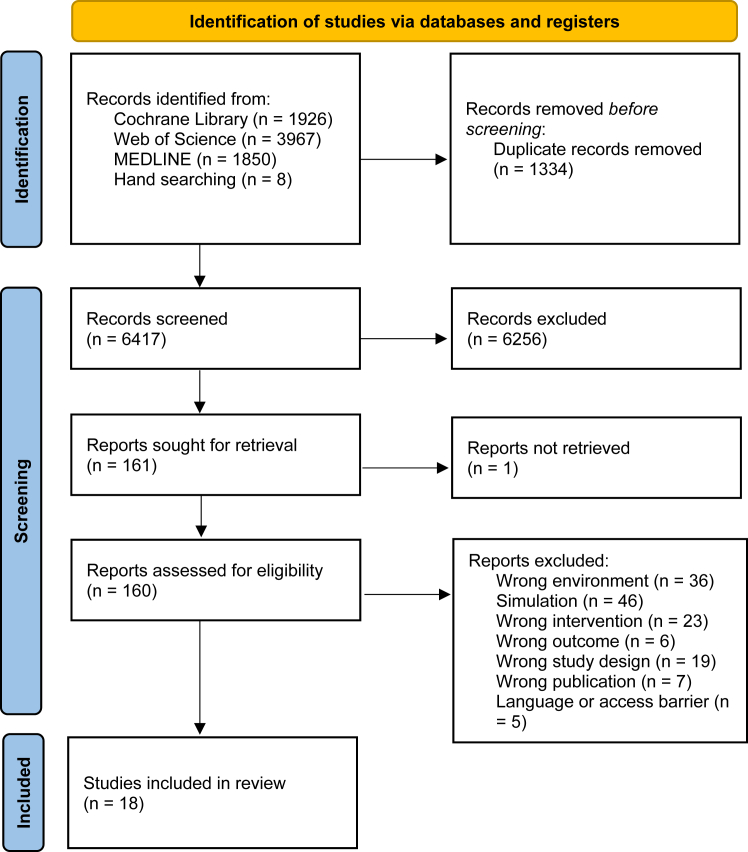
PRISMA flow diagram.

The initial search yielded 6417 articles (after duplicates were removed), 6256 articles were excluded as these did not meet the inclusion criteria outlined above, one article was removed at this stage as it could not be retrieved. We reviewed 160 articles at the full text stage and excluded 142 because they did not describe aerosol concentration, were not carried out in a hospital setting, were a simulated model, or were excluded study designs such as reviews, or were not a peer reviewed article. 18 articles were included in the review (see Fig. 1 for the PRISMA Flow Diagram).

### Article characteristics

Main article characteristics are summarised in Table 1. Four articles were from the UK, three from the USA, two from Australia, two from China, and one article from each of the following countries: Hong Kong, Ireland, South Korea, Germany, Singapore, and Iran. One study was also conducted among a few Eastern European countries.

**Table 1:** Study characteristics.

Authors; country;MMAT score	Aims of study	Type of intervention	Healthcare setting and environment	Health condition	Main findings
Conway Morris et al.^13^ UK MMAT: 4/5	Assess the removal of airborne SARS-CoV-2 in a hospital environment using combined air filtration and Ultra Violet (UV) sterilisation technology.	AC1500 High Efficiency Particulate Air (HEPA)14/UV steriliser (ward) and Medi 10 HEPA13/UV steriliser (ICU). No detail on UV wavelength. One air filter was placed in the corner of each ward at a fixed position, switched on, and run continuously for 24 h for 1-week, providing approximately 5–10 room-volume filtrations per hour.	Surgery ward (4-bedded) and ICU (5-bedded). Both were fully occupied.	COVID-19 (based on identification of SARS-CoV-2 RNA in air samples).	Able to detect airborne SARS-CoV-2 RNA in a repurposed COVID-19 ‘surge ward’ prior to use of a filter, following filtration SARS-CoV-2 RNA was not detected. SARS-CoV-2 was infrequently detected in the air of a ‘surge ICU’ even prior to filtration, despite this the filtration device retained its ability to reduce microbial bioaerosols significantly (p = 0.05).
Butler et al.^14^ UK MMAT: 2/4	To assess aerosol transport within the ward and determine whether the air cleaning unit (ACU) reduced airborne particulate matter (PM) levels.	The ACU was a hybrid system that combined HEPA filters and UV-C lamps (at 254 nm), and had a clean air delivery rate of 2550–3000 m^3^/h. The single ACU was positioned opposite the two six-bedded patient bays.	Half a ward on sixth floor of hospital, three side rooms each with a door and two 6-bedded bays open to a central corridor. Ventilated by a central mechanical ventilation system and openable windows, with the bed bays and side rooms positively pressurised with respect to the central corridor. The ‘open/closed’ status of the various windows and doors on the ward was not recorded. Ward ventilation rates ranged from 1.7 to 5.8 (median 4.0) air changes per hour.	Respiratory viruses (based on PM as a simulation for removal of infectious aerosols).	Particles up to 10 μm travelled considerable distances around the ward, and the ACU reduced particulate matter (PM) levels of all sizes throughout the space, not just near the device. All results were strongly significant (p < 0.001).
Lu et al.^15^ China MMAT: 1/2	1) The purpose of this study is to analyse the environmental parameters of Chang Gung Memorial Hospital, to clarify the environmental characteristics of fever clinics during the COVID-19 epidemic. 2) To establish a control method of hospital infection in fever clinics combined with epidemic prevention, combined with the hospital's control measures for patients' behaviour.	Natural ventilation based on opening external doors and windows. Indoor monitoring equipment is set up in the hospital to measure carbon dioxide concentrations.	Fever clinic containing the outpatient nursing station, doctors office, and corridor of waiting area.	COVID-19 (based on CO_2_ as a simulation for removal of SARS-CoV-2).	Daily CO_2_ concentration in nursing station, doctor's office, and corridor of the waiting area of the hospital was lower than the theoretical limit, and during the period when the patients with confirmed COVID-19 stayed in the fever clinic, daily CO_2_ in the corridor of the waiting area was lower than the theoretical limit. The natural ventilation was good, meeting indoor ventilation dilution level required by infectious disease hospitals. Statistical significance not assessed.
Mousavi et al.^16^ USA MMAT: 3/4	Determine the effectiveness and ideal placement of portable HEPA units. Evaluate the effectiveness of negative pressurisation, as well as a temporary anteroom structure on minimising the dispersion of contaminants in the hospital space.	Two portable HEPA machines (Abatement Technologies PAS2400) equipped with brand new HEPA filters. The two machines established various pressurisation schemes across the plastic barrier and the main door. When turned on, the HEPA machines drew air at a 1500 m^3^ h-1 rate and discharged the filtered air to the outdoors. One HEPA machine produced 20 air changes per hour (ACH) in the entire room (i.e., ISO + ANT).	Patient room that was decommissioned was used and was 6.3 m (L) × 3.9 m (W) × 3.0 m (H) and connected to the hallway that had a wood door that was sealed from other adjacent spaces with block walls and drywall ceilings. A temporary plastic barrier was installed inside the room, 4.0 m away from the entrance to the patient room and divided the room into an isolation room (ISO) and an anteroom (ANT).	COVID-19 (based on aerosol particles as a simulation for removal of SARS-CoV-2).	Aerosol particles were generated using an oil-based substance and a pharmaceutical nebuliser connected to an air pump. Particles sized 0.3 μm were measured as they were most similar to the size of SARS-CoV-2. When HEPA was present, isolation room (ISO) concentration was reduced until the next aerosolization indicating effectiveness of HEPA filtration. There was no control over particles that could escape the room, and significant accumulation of particles was observed when the HEPA machines were both off. Statistical significance not assessed.
Li et al.^17^ Hong Kong MMAT: 1/2	Investigate the ventilation of the wards after 18 months of operations and identify the major factors that affect ventilation effectiveness.	Pressure difference, airflow direction through doorways, air change rate, and local ventilation effectiveness.	Nine hospitals with many new SARS wards were selected. The SARS wards were designed to maintain negative air pressure to avoid outward air flow from the ward into adjacent areas.	SARS 2003 Epidemic (based on smoke as a simulation for air flow and air leakage).	When the door was closed in the low pressure rooms, all tests showed inward airflow, suggesting SARS wards were effective at preventing leakage of air from the low pressure ward into the adjacent corridors. The findings that the local ventilation effectiveness is not uniform in all tested cubicles indicated that the air in these rooms is not well mixed. Statistical significance not assessed.
Oberst & Henrich^9^ Germany MMAT: 4/5	To determine whether the presence of a filter in a consultation room can reduce airborne transmission.	HEPA filter (H13 – European Norm1822, efficiency 99.95% of 0.3 μm/m per h) with plasma and UV-light radiation, located near the desk, between the examiner and patient chairs. No detail on UV wavelength. The number of particles were recorded at 15-min intervals. As a comparison, the measurement was carried out the previous day without using the air filter device.	Consultation room in an orthopaedic clinic (room volume 52 m^3^).	COVID-19 (based on PM as a simulation for removal of SARS-CoV-2).	Use of filter led to a reduction in PM2.5 by over 50% compared with absence of the filter. Statistical significance not assessed.
Fennelly et al.^18^ Ireland MMAT: 3/4	To compare the effectiveness of natural ventilation and HEPA filtration, alone and in combination for clearing aerosols from a legacy design ward bay using continuous measurements of airborne particles.	Natural ventilation and HEPA filter (H13) device (CC2000, Camfil, Ireland) that was placed against the right wall of the bay, 1.5 m from the door. Air intake was from both sides of the device parallel to the wall, and filtered air was expelled forwards into the room. Four tests were performed under different ventilation conditions (‘windows open, air filtration unit (AFU) on’, ‘AFU alone’, ‘windows alone’, and ‘windows closed, AFU off’). The AFU was operated at half capacity corresponding to the manufacturer-claimed air passage rate of 480 m^3^/h.	6 bed legacy ward bay (171 m^3^ room volume). Entrance door sealed with a polythene barrier, and three windows on one side. There was no heating, ventilation and air conditioning system for air handling. The hospital weather station data gave wind speed of 2.6–5.1 m/s from east-SSE.	COVID-19 (based on PM produced from nebuliser as a simulation for removal of SARS-CoV-2).	All ventilation types were successful in reducing PM2.5 concentrations, and the portable air filtration unit (AFU) successfully augmented natural ventilation of airborne particles. The ‘windows open, AFU on’ produced the lowest concentrations and highest clearance rate of PM2.5. The ‘windows alone’ condition was unable to reduce concentrations back to baseline levels without aid of the AFU. The mean PM2.5 clearance rate was significantly (p < 0.01) higher in the ‘windows open, AFU on’ condition compared with the ‘AFU alone’ condition, which, in turn, was significantly higher than the ‘windows alone’ condition.
Vokurka et al.^19^ Eastern Europe MMAT: 3/5	To establish whether HEPA filtration was available within central and eastern European transplant centres and to obtain data about its impact on the incidence of pneumonia and mortality up to day 100 in patients after autologous and allogeneic HSCT.	HEPA – data was collected on each transplant unit including whether they had a HEPA filter.	An observational, prospective study was performed on haematopoietic stem cell transplantation (HSCT) patients across nine transplant units.	Pneumonia defined as elevated body temperature and/or CRP, with radiology imaging findings.	Autologous HSCT group: In respect to pneumonia incidence, there was no statistically significant impact of HEPA filtration presence or absence (p = 0.73) observed within the group. No differences in mortality up to day 100 post-transplant: 4.5% in HEPA vs. 4.9% in Non-HEPA -filtered rooms (p = 1.0). Allogeneic HSCT group: Pneumonia incidence - no impact of HEPA filtration presence or absence (p = 0.09) observed in this group. The pneumonia incidence in HEPA-filtered rooms was 18.254 (7%) vs. 6/35 (17%) in non-HEPA-filtered rooms (p = 0.05). There were no differences in mortality up to day 100 post-transplant: 14% in HEPA vs. 17% in Non-HEPA-filtered rooms (p = 0.6).
Buising et al.^20^ Australia MMAT: 3/4	To study the airflow, transmission, and clearance of aerosols in the clinical spaces of a hospital ward that had been used to care for patients with coronavirus disease 2019 (COVID-19) and to examine the impact of portable air cleaners on aerosol clearance.	Air cleaners were domestic appliances (Samsung AX5500K) equipped with H13 HEPA filters capable of filtering 99.97% of particles at a clean air delivery rate of 467 m^3^ per h. Two air cleaners were placed along the bedside and at the foot of the bed. Measurements were taken concurrently in the single-patient room and at the nurses' station at 10-s intervals until the aerosols cleared.	Empty ward previously used to care for COVID-19 patients. Ward had a long central corridor and 11 rooms, which usually accommodates 25 patient beds (4 single rooms with en suite bathrooms and 7 three-bed shared rooms, each with a shared en suite bathroom). No rooms in the ward have negative pressure, the ward has its own closed, ducted Heating, Ventilation, Air Conditioning (HVAC) system, that delivers 12 air changes per hour. No windows in the ward can be opened, and the return air vent for the whole ward is above the single entrance and exit point to the ward (just inside the door to the ward). Rooms all have doors with a small gap at the bottom (∼5 cm) to allow air egress. One of the single-patient rooms with a room floor space of 12.8 m^2^ and volume of ∼37 m^3^ was selected for the study. The corridor outside the room was ∼2 m wide, and the patient room was directly opposite the open nurses' station, which had a front desk with entrance spaces on either side.	COVID-19 (based on aerosol smoke particles as a simulation for removal of SARS-CoV-2).	Two air cleaners in the patient's room with the door closed or open, the room cleared of 99% of all aerosols in 5.5 min (67% reduction) compared to no air cleaners. At the nurse's station, the smoke cleared more quickly in <3 min. Statistical significance not assessed.
Park et al.^21^South Korea MMAT: 3/4	To investigate ventilation strategies to prevent nosocomial transmission of COVID-19.	Ventilation strategies. The airflow around the ward on the 8th floor of the main building was visualised, and the diffusion characteristics of the particulate matter were analysed. The diffusion characteristics of the particle matter were measured under the following conditions: windows closed; patient room door opened; windows and patient room door closed; and windows open and patient room door open.	Study institution was a 725-bed referral-teaching hospital. 13 wards. The main building consisted of 10 floors. Each floor had 58–70 beds. Among them, 96.2% (304/316) were multi-bed rooms and 90.2% (285/316) were five- or six- patient rooms. Two HVAC systems were installed in the main building with a capacity of air change rate of a supposed 6 × per hour in the ward; the actual supply and exhaust air volume on 8th floor ward was 1.44 × per hour on average.	COVID-19 (based on oil-based PM as a simulation for airflow of SARS-CoV-2).	Opening windows and closing the door to the patient room allowed for natural ventilation and minimised the spread of particles to adjacent rooms. Statistical significance not assessed.
Ryan et al.^22^ USA MMAT: 4/5	To test the hypothesis that enhanced ultraviolet germicidal irradiation (eUVGI) installed in a neonatal intensive care unit (NICU) HVAC would decrease ventilator associated-pneumonia (VAP) and neonatal intensive care unit (NICU) environment microbes.	Enhanced ultraviolet germicidal irradiation (eUVGI) in HVAC system. No detail on UV wavelength.	NICU supplied a HVAC system. Several practices remained unchanged throughout the study period including: (1) infection control protocols for hand washing and universal contact precautions; (2) the NICU surface cleaning schedule and materials; and (3) respiratory protocols for equipment cleaning, ventilator circuit changes and daily humidifier water changes.	Ventilator associated-pneumonia (VAP).	At baseline, the HVAC components were visibly contaminated. By approx. 6 weeks the HVACs had no visible contamination by microbes and by 6 months HVAC cultures were negative. NICU surface cultures approached zero during enhanced ultraviolet germicidal irradiation (eUVGI) (p < 0.0001). After eUVGI was installed in HVAC, VAP decreased in the high-risk sub-population of infants from 74% to 55% after 6 months and to 44% at 18 months (p = 0.04).
Salam et al.^23^ Singapore MMAT: 5/5	To assess the impact of 48 portable HEPA filter units deployed in selected wards in Singapore General Hospital, an acute tertiary-care hospital in Singapore.	HEPA filter.	Six wards that cater to different needs of patients, all fitted with a HEPA filter.	Invasive aspergillosis.	In the wards in which portable HEPA filters were deployed, the incidence of invasive aspergillosis (IA) of 34.61/100,000 patient-days during the preinstallation period decreased to 17.51/100,000 patient-days during the post-installation period (p = 0.01). Using all cases of proven, probable and possible IA, the risk of acquiring IA was significantly lower in the presence of portable HEPA filters, adjusted for presence of an immunosuppressive condition. Patients who were admitted to these wards after installation of portable HEPA units had ∼51% lower risk of acquiring IA.
Rezaei et al.^24^ Iran MMAT: 1/2	To establish a novel technique for eliminating SARS-CoV-2 from cleanrooms HVAC systems using the recovered heat of exhaust air.	HVAC – the system consists of three main components, namely, an outdoor air intake and air exhaust ducts and controls, an air handling unit (AHU), and air distribution systems. An air handling unit by itself is composed of a HEPA filter, a humidifier, a cooling/heating coil, and ultraviolet light emitters. No detail on UV wavelength. The proposed system has a mechanism to warm the exhausted air, which should eliminate SARS-CoV-2 aerosols.	Hospital air cleaning room.	SARS-CoV-2	The temperature and relative humidity limits of the exiting air are reported to be in the range of 50–80 °C and 40–50% respectively. The study can conclude that under such conditions, SARS-CoV-2 should be eliminated. Statistical significance not assessed.
Lee et al.^25^ Australia MMAT: 3/4	To assess the effectiveness of aerosol filtrations by portable air cleaning devices with high-efficiency particulate air filters used in addition to a standard building HVAC unit.	Two portable air cleaning devices (Air Purifier AX60RR5080WD, Samsung Electronics, Seoul, South Korea). The flow rates (i.e., CADR) of the HEPA filters used in the hospital room was 467 m^3^/h and was equipped with standard HEPA filters capable of filtering 99.97% of particles >0.3 μm. The air cleaning devices were placed in regions that were close to a hospital bed and suspected to have poor air circulation by inspection.	Single-bed hospital room (room volume 37 m^3^) with HVAC system with 13.9 air change per hour (ACH).	COVID-19 (based on aerosol smoke particles as a simulation for removal of SARS-CoV-2).	Hospital room with a HVAC alone had a relatively high flow rate at baseline (13.9 ACH), but, when there were two air cleaning devices in the room (39.2 ACH in total), the clearance time was significantly improved to three times faster (<10 min). Statistical significance not assessed.
Rao et al.^26^ USA MMAT: 5/5	To assess whether portable photo electrochemical oxidation (PECO) air purification in the paediatric hospital setting could improve health outcomes for patients admitted with respiratory distress.	Portable PECO-equipped portable air purifier devices. The units were placed as close to the patient's breathing zone as safely possible with appropriate safety precautions. Units were placed in 20 private rooms and in 7 paediatric intensive care unit (ICU) rooms. Staff members were trained to operate the devices and to ensure that they were running 24 h a day. If patients were bothered by the unit's sound or light, staff were trained in turning off the devices.	20 private rooms and in 7 paediatric ICU rooms.	Respiratory distress (based on impact of PECO air purification on hospital stay and reliance on ventilation equipment).	Rate of non-invasive ventilation use was 77% in the pre-intervention period and 23% in the post-intervention period. The decrease in non-invasive ventilation use in the pre-intervention cohort compared with the post-intervention cohort was statistically non-significant. Rate of nebuliser use was 59% in the pre-intervention period and 41% in the post-intervention period. Decrease in nebuliser use in the pre-intervention cohort compared with the post-intervention cohort was statistically non-significant. Rate of intubation was 57.1% in the pre-intervention period and 43% in the post-intervention period. Decrease in rate of intubation in the pre-intervention cohort compared with the post-intervention cohort was statistically non-significant.
Salmonsmith et al.^27^ UK MMAT: 3/4	To investigate the effect of using portable air cleaners, a low-energy and low-cost alternative, to reduce the concentration of aerosols in typical patient consultation/procedure environments.	Portable Air Cleaners (PAC). A smaller unit (Core 200S Smart True HEPA Air Purifier, Arovast Corporation, CA, USA); and a larger unit (LV-H133 Tower True HEPA Air Purifier, Arovast Corporation). When switched on, both devices were set to medium the smaller resulted in a flow rate of 1.1 m^3^/min (equivalent to a CADR of 68.9 m^3^/h) and the larger 2.2 m^3^/min (equivalent to a CADR of 155.3 m^3^/h). Medical professionals indicated that the high flow setting was too noisy to be used during all conversations with patients, the medium flow setting was acceptable.	3 rooms: A laboratory room at UCL with inlet and outlet air ventilation panels. Two rooms in the National Hospital for Neurology and Neurosurgery. One room was a consulting room and the second was a procedure room. Neither room had ventilation and represented rooms in most old hospitals within the NHS. The first room was a consulting room (R2), measuring approximately 3.2 m × 4.7 m × 2.6 m, with no ventilation panels; and the second was a procedure room, measuring approximately 4.1 m × 4.8 m × 2.5 m, with no ventilation panels.	COVID-19 (based on saline aerosols as a simulation for removal of SARS-CoV-2).	Portable air cleaner mitigation is very effective in cleaning the air of aerosols in these rooms with minimal other sources of air change. Correct use of PAC can reduce the half-life aerosols by 82% compared to the same indoor-environment without any ventilation and at an equivalent rate to built-in mechanical ventilation. The highest level of aerosol concentration measured when using PAC remains at least 46% lower than that when no mitigation is used. Statistical significance not assessed.
Li et al.^28^ China MMAT: 1/2	To systematically analyse the presence of airborne SARS-CoV-2 in the isolation ward and within the contaminant, emergency, and clean zones of a designated COVID-19 hospital.	Closed-loop of the contaminant, emergency, and clean zones, allowing only the one-way flow of people and materials from the clean zone to the contaminant zone. All isolation wards were normally functioning under negative pressure.	Air samples were collected from spaces in the closed-loop zone of Beijing Ditan Hospital of the Capital Medical University. The contaminant zone was a high-risk space and was located near the isolation ward. The emergency zone was adjacent to the contaminant zone and had a single entrance into the contaminant zone and an exit out of the contaminant zone through the first and second rooms for taking off PPE. The clean zone was only accessible to medical staff and was lateral to the emergency zone.	Detection of movement of SARS-CoV-2.	The SARS-CoV-2 negative results of air samples collected in the clean and emergency zones demonstrated the existing measures to interrupt virus transmission in designated COVID-19 hospitals (closed-loop management, unidirectional airflow, and negative-pressure wards). A total of 359 air samples collected in the emergency and clean zones tested negative for SARS-CoV-2 after 20 days of monitoring. However the results showed that even under negative pressure ventilation, the airborne SARS-CoV-2 could still leak out of isolation wards when the door was opened during physicians' daily rounds and meal deliveries. Statistical significance not assessed.
Otter et al.^29^ UK MMAT: 3/4	To conduct an evaluation of the technical specification of HEPA-based air disinfection systems currently available on the UK market.	HEPA filters: Unit A was Rediair H14 (Gama Healthcare Ltd.), Unit B was Rensair H13 (QO1B, RensairLtd.), and Unit C was AirSentry H14 (AirSentryLtd.). Rensair running at 356 m^3^/h airflow; Rediair running at 622 m^3^/h airflow; Air Sentry running at 1228 m^3^/h airflow. The ability to remove smoke released at various points in the room was tested using a smoke generator (ConceptAir Trace, ConceptSmoke). The impact on particle counts was measured at several points in the room using a particle counter (TROTECPc220).	Unoccupied hospital room with a volume of 38 m^3^.	Air based pathogens (based on aerosol smoke particles as a simulation for removal).	Particle count testing showed that the higher the setting, the more impact on particles that could be identified in the room, with a 30% (standard deviation 20%) reduction on 0.3 μm particles achieved by Rensair running at 356 m^3^/h, 60% (standard deviation 11%) reduction by Rediair running at 622 m^3^/h, and 81% (standard deviation 10%) reduction by Air Sentry running at 1228 m^3^/h. Smoke testing showed the units were able to pull in air from most parts of the room, although the inclusion of objects that interrupted airflows meant that smoke was removed less effectively. Statistical significance not assessed.

Several different forms of evaluation were conducted. This included pre- and post-designs that assessed the impact on the concentration of particles or microbial components or conditions of people in the room with no intervention compared to then introducing the intervention (or vice versa) or with different interventions.^9^^,^^13^^,^^14^^,^^16^^,^^18^^,^^20^, ^21^, ^22^, ^23^^,^^25^, ^26^, ^27^^,^^29^ There were evaluations comparing the concentration of particles in the room with a theoretical limit based on standards^15^ and evaluations comparing populations in different wards who had access to an intervention vs. no intervention.^19^ Also conducted were evaluations that measured airflow, air change, pressure difference, ventilation effectiveness or temperature when interventions were present, however there was no comparison.^17^^,^^24^^,^^28^

The studies were evaluating the impact of interventions on a range of outcomes such as outcomes in human participants,^19^^,^^22^^,^^23^^,^^26^ outcomes based on microbial components or aerosols emitted by humans,^9^^,^^13^^,^^22^^,^^24^^,^^28^ and aerosols mimicking those emitted from humans.^14^, ^15^, ^16^, ^17^, ^18^^,^^20^^,^^21^^,^^25^^,^^27^^,^^29^ Statistical significance was assessed in seven of the studies.^13^^,^^14^^,^^18^^,^^19^^,^^22^^,^^23^^,^^26^ Very few articles discussed if there were changes to external particulate matter (PM) other than changes to the aerosols or particulate matter being produced and measured for the research. Butler et al. did flag limitations in their research where windows and doors were opened but not recorded, so external PM may have affected their measurements.^14^ Lu et al. discussed outdoor CO_2_ concentrations near the hospital and flagged these fluctuated due to traffic and weather.^15^

### Thematic areas

The articles outlined interventions that are currently used to improve aerosol concentrations in hospitals all of which have been evaluated to some degree. The findings can be grouped into three themes. The first theme includes findings around the filtration and inactivation of aerosol particles; the second theme discusses airflow and ventilation; and the third theme is around improvements or reductions of health conditions because of interventions.

#### Filtration and inactivation of aerosol particles

Eleven of the studies discussed the importance of filters such as HEPA and air cleaning units (ACUs) containing filters, and heating, ventilation, air conditioning (HVAC) systems that were found to reduce the amount of aerosol particles in the air.^9^^,^^13^^,^^14^^,^^16^^,^^18^^,^^20^^,^^24^^,^^25^^,^^27^^,^^29^

Conway Morris et al. found that previously detected SARS-CoV-2 within a COVID-19 ward was no longer detected after use of a HEPA filter but was again detected following removal of the filter.^13^ Butler et al. found that particles up to 10 μm travelled considerable distances around a ward (beyond 2 m), however, the ACU reduced the PM levels throughout the space (not just near the device).^14^ Oberst and Heinrich similarly reported that the addition of a filter into a consultation room could significantly reduce the risk of airborne transmission, with aerosol concentration of PM2.5 decreasing by a minimum of 50%.^9^ Otter et al. demonstrated that the use of three different HEPA filters could lead to a 30–81% reduction of 0.3 μm smoke particles.^29^ Mousavi et al. found a reduction in particle concentrations when HEPA machines were present, and a significant accumulation of particles observed when the HEPA machines were both off.^16^ Fennelly et al. suggested that air ventilation (open windows) alone was unable to reduce concentrations back to baseline levels without the aid of an air filtration unit (AFU), with the ‘windows open, AFU on’ condition producing the lowest concentrations and highest clearance rate of PM2.5.^18^ Salmonsmith et al. reported that portable air cleaner (PAC) mitigation is very effective in clearing the air of aerosols in rooms, they also noted PAC reduction of the half-life of aerosols by 82%.^27^

Rezaei et al. looked at cleaning rooms, they identified that a HVAC system provided exhaust air ranging from 50 to 80 °C and with 40–50% humidity, under these conditions COVID-19 should be rapidly eliminated.^24^ Conversely, Buising et al.^20^ found that the existing ward HVAC system alone was inefficient when clearing a patient room of aerosols and that use of HEPA filters improved clearance. Lee et al.^25^ had similar findings that the use of air purifiers were able to speed up the clearance of air particles compared to the use of a HVAC system alone.

#### Effect of ventilation and airflow on aerosol concentration

Findings related to ventilation and airflow changes varied as Lu et al.^15^ showed how relying on ventilation through open windows and doors could be beneficial to keep CO_2_ within specific limits, but Li et al.^28^ showed how doors could negatively impact the efforts of negative pressure systems and unidirectional airflow in keeping SARS-CoV-2 contained within separate zones of a ward. Li et al. was however able to demonstrate the ability of unidirectional airflow and negative-pressure wards in interrupting virus transmission through samples of air in the clean zone and emergency zone as there was no SARS-CoV-2 in these areas.^28^

Another study on the conditions of a sealed ward designed to maintain negative air pressure and avoid outward air flow, by another Li et al., tested air movement and found, when the door was closed, all tested wards had inward airflow or there was no outward airflow, suggesting that these new SARS wards are effective in securing no-leakage of cubicle air into the corridors, even when some of the cubicles failed to maintain a negative pressure difference.^17^ Park et al. also highlighted the effectiveness of natural ventilation, and found that opening windows minimised the spread of particles to adjacent rooms compared to relying on a ventilation system alone.^21^

#### Improvements or reduction in health conditions

Only four of the articles looked at the direct impact of interventions on health conditions (pneumonia^19^^,^^22^ invasive aspergillosis,^23^ and respiratory distress^26^). Many of the other publications often used simulations of particles that mimicked the infectious agents that cause health conditions. All four articles outlined improvements in health conditions as a result of an intervention (enhanced UV germicidal irradiation in HVAC systems, HEPA filters, PECO air purifiers) through changes in aerosol concentrations, despite only two of the publications having statistically significant results. None of the publications reported what impact seasons may have had on the changes in health conditions. Two of the studies had durations for over one-year^22^^,^^23^ and one study was run from August to December.^26^

Ryan et al. reported after using an enhanced UV germicidal irradiation (UVGI) in HVAC systems, ventilator-assisted pneumonia (VAP) was decreased from 74% to 55% after 6-months (January 2002) and to 44% after 18-months (January 2003) (p = 0.04). However, it is worth noting that these findings are only relevant to HVAC systems which recirculate air, this is rare in UK healthcare settings as this method does not provide the level of filtration needed for hospitals.^22^ Additionally, the study did not evaluate if there were any by-products related to the use of UVGI. Salam et al. found that after HEPA filters were installed, incidences of invasive aspergillosis (IA) significantly decreased, individuals admitted after the HEPA filters were installed had around 51% lower risk of acquiring IA.^23^ The change in incidence was calculated over the 31-month study period. This study however did not report on the potential mechanism behind the HEPA filter's impact such as changes to air flow.

The two publications that found non-significant improvements in health conditions as a result of interventions stressed however that the results were still clinically meaningful. Rao et al. found improvements after the implementation of portable PECO air purifiers when running the study between August and December 2018. Pre-to post-intervention for patients showed non-invasive ventilation improved from 77% to 23%, rate of nebuliser use from 59% to 41% and rate of intubation from 57.1% to 43%.^26^ Whilst Vokurka et al.^19^ found an almost significant trend for reductions in pneumonia in the presence of HEPA filters, and therefore recommended where possible to use HEPA filters in rooms with immunocompromised patients.

### MMAT findings

Details on each publication's MMAT scores can be found in Table 1 and in Appendix S4. This appraisal tool is mainly used to assess studies with human participants, so there were questions not applicable for some of the included studies. There were only six studies that seemed relevant to assess against the entire MMAT criteria, one of the studies had a medium quality ranking (2/5 or 3/5) and five had a high-quality ranking (4/5 or 5/5).

The remaining 12 studies were based on simulations of infectious aerosols, and therefore there were items that were non-applicable. We therefore assessed these studies out of a score of two or four. One study reached 2/4, seven studies reached 3/4 and four studies reached 1/2. However, we recognise limitations in ranking the simulation studies as many of the criteria they were assessed against were not applicable.

## Discussion

The aim of this rapid evidence review was to identify interventions being used in hospitals in an attempt to improve aerosol concentrations through filtration, aerosol inactivation, air flow and ventilation. Overall, we found that ventilation involving the exchange of old air for fresh air needs to be improved to support the organic process of preventing airborne transmission.^15^^,^^17^^,^^21^ The included studies have primarily focused on how air filtration and ventilation have and could impact transmission of infectious airborne particles. Ventilation and filtration are each just one of the many approaches that can be used to improve air quality in medical settings and must not be considered siloed from other factors such as microclimates, room dimensions, use of chemical products etc.^8^^,^^30^, ^31^, ^32^ These actions must be part of an organic strategy aimed at continuous improvement of air quality.

The studies observed the impact of air filtration focussing on the removal of certain particles from the existing air. Filtration was the most commonly discussed intervention, and was shown to be effective in multiple studies. Butler et al. measured PM levels throughout a ward before and after activation of a HEPA/UV-C air-cleaning unit and found its activation to significantly reduce PM levels. Additionally, these levels were reduced throughout the ward, not solely near the device.^14^ Mousavi looked specifically at optimising the location of portable air purifying units and found that it is best placed near the patient's bed.^16^ Similarly, Conway Morris et al. detected COVID in the air before activating their HEPA/UV-C unit, but not once it was in use,^13^ Oberst also came to the same conclusion.^9^ All the findings suggest portable air filtration devices can improve patient and healthcare worker safety by reducing airborne transmission. This suggestion was supported by the finding that transplant patients treated in HEPA-filtered rooms experienced lower incidences of pneumonia than those in rooms without HEPA filtration.^19^ Three different devices led to improvements or reductions in health conditions, ventilator-assisted pneumonia decreased after the implementation of an eUVGI in a HVAC system,^22^ invasive aspergillosis incidences decreased after installing a HEPA filter,^23^ reductions in cases of pneumonia in wards that had HEPA filters,^19^ and decreases in non-invasive ventilation, nebuliser use and intubation after using a portable PECO air purifier, although these were not all statistically significant.^19^^,^^26^

In addition to their clinical benefits, HEPA filters are easy to deploy and cost effective, despite this, they do little to improve the air flow in hospitals, but rather mitigate infection risk from PM. The authors that examined the efficacy of ventilation called for implementing air purifiers as natural or mechanical ventilation may not be sufficient to prevent transmission.^21^ Based on these findings, and in relation to existing research it is recommended to include air purifiers within a formal air quality and management strategy.^33^^,^^34^

Along with potential positive impacts from the ventilation and filtration approaches it is important to note any barriers in implementing the approaches such as noise pollution as was reported by Rao et al. and Otter et al.^26^^,^^29^ or adverse health events from using devices (e.g., the impact of UV radiation or the potential for free-standing devices to fall and cause injury). Very few of the studies discussed the potential adverse impacts of the devices on relative humidity, CO_2_ or ozone generation as has been reported previously.^35^ Conway et al. didn't measure this but flagged that further research would be needed on the effect of HEPA filters on reducing ambient humidity.^13^ Butler et al. actually found that CO_2_ levels and vapour pressure reduced in the presence of the ACU but couldn't attribute why this was and thought it may have been due to the unrecorded opening of windows and doors.^14^

NHS England guidance recommends that devices containing HEPA filters used within the healthcare setting are classified as BS EN1822-1 or ISO 29463-1. This means the HEPA filters have been tested and met the efficiencies for 99.95% (H13 filter) and 99.995% (H14 filter).^36^^,^^37^ Only one of the publications in this review specifically referenced EN 1822,^9^ and some shared that the HEPA filters were of the class H13 or H14^13^^,^^18^^,^^20^^,^^29^ or that they were capable of filtering 99.97% of particles >0.3 μm.^25^ Many of the included publications have referenced the Clean Air Delivery Rate (CADR), which helps to build a better picture about the devices that have been used.^14^^,^^16^^,^^18^^,^^20^^,^^25^^,^^27^^,^^29^ The CADR is used by manufacturers to demonstrate how fast the device removes a specific size of particles from a test environment, however it does not demonstrate how it will function in reality, when room size, number of people in a room and background ventilation are at play.^37^ A few of the publications have provided detail on the realities of using such devices for instance that with time the effectiveness of such devices reduces^16^^,^^22^ and that some of the devices were operating at half of the capacity the manufacturer had claimed.^18^^,^^21^ Some also shared how much it had cost to install and maintain devices,^23^^,^^29^ others shared how frequently the filters were changed^22^ and how staff had been trained to operate devices.^23^^,^^26^ The necessity to maintain such devices to ensure they are functioning correctly is essential and must be considered when installing, in addition to any barriers that may occur in ensuring their maintenance such as the economic cost, and time it takes to fulfil these activities.

## Strengths and limitations

Time restrictions, search terms and the number of databases searched allowed for a rapid review of the existing literature, however it does limit the data that was available to us, particularly as time constraints did not allow for grey literature searching. A small but well-rounded number of databases and relatively specific search terms were used in order to identify specific articles and facilitate rapid screening. These are key features of rapid reviews allowing for swift reports, although only three databases were searched they are central databases for the research conducted. The review was also limited as no protocol was published prior to commencing the research, instead we had an internal proposal document to keep us to account and to guide us with the research. This review was also strengthened by having four reviewers searching for articles and cross-checking the relevance of peer-reviewed articles. Due to the heterogeneity of the literature regarding the interventions delivered and outcomes assessed, we were again limited as we were unable to conduct a meta-analysis of the data. The MMAT was used to assess the quality of the included publications, however it was not possible to implement all the critiera for the studies that did not include human subjects, making it difficult to be certain of their quality.

## Outstanding questions and need for further research

Many of the studies investigated the effect of interventions on simulated aerosols, further research is needed to assess its effect on real viral particles such as SARS-COV-2^9^ and measuring the presence or absence of infection in healthcare professionals and patients as an outcome.^13^ There is a need to consider the potential harm of adding air purifiers to medical wards, through effects on noise, reduced ambient humidity, and impact on the delivery of care.^9^^,^^13^^,^^29^^,^^35^ Existing literature has also identified health and safety risks of using UV sterilisation in the proximity of people, and has called for additional research into how hospital ward characteristics (humidity and temperature) can impact the effectiveness of the technology.^38^ Whilst our search strategy did not encompass techniques to manage aerosol concentrations such as heat and internal pressure difference, these approaches were still discussed in the included literature and were found to be effective at mitigating aerosol concentrations.^17^^,^^24^^,^^28^ Heat, internal pressure difference, microclimates, use of chemical products, room size, and number of people or type of furniture in a room, are avenues to research further in terms of their impact on air quality and must be considered as a multi-pronged approach as the factors all interact with each other.

There are numerous methods currently being used in hospitals to improve aerosol concentrations through ventilation, airflow, filtration, and pressurised rooms. Papers identified that the air change rates currently in use were not frequent enough to ensure effective reduction of infectious aerosols and that reliance on some of the above interventions would support with this. However the barriers to using such approaches must be considered in addition to ensuring their efficiency is monitored regularly. The articles also outlined the importance of aerosol concentrations in hospitals to reduce infections, identifying that using eUVGI in HVAC systems, HEPA filters and PECO air purifiers improved patient outcomes. Finally it is of huge importance that efforts to improve aerosol concentrations are considered as part of an organic strategy that encompasses a huge range of factors in addition to ventilation and filtration (microclimates, room dimensions, use of chemical products etc.) that can facilitate continuous improvement of air quality.

## Contributors

C.V.P. and L.B.L. designed the study. G.B. drafted the paper with input from all authors. S.E.C. revised the paper with input from all authors. G.B. performed the searches and S.E.C. performed updated searches. G.B., R.D.K., F.B., and S.E.C. conducted data extraction and analysis. G.B., S.E.C., F.B., and C.V.P. accessed and verified the underlaying data. Authors responsible for the review and editing of the manuscript included: L.B.L., C.V.P., G.B., F.B., R.D.K., S.E.C., M.K.T., S.W., A.D., R.T., D.M., E.L.D., J.S., F.S., Z.K., H.M.D., L.C., R.E., J.R., C.F.H., and R.H.

## Data sharing statement

Data collected for this review will be shared upon request.

## Declaration of interests

L.B.L. declared funding from an NIHR PGfAR grant (payment through institution); declared support from the National Institute for Health Research University College London Hospitals Biomedical Research Centre and the Wellcome/EPSRC Centre for Interventional and Surgical Sciences (WEISS); and declared a patent planned.

C.V.P. declared funding from an NIHR grant.

S.E.C. declared funding from an NIHR grant.

F.B. declared funding from University College London.

R.H. declared funding from grants and consultancy fees, and declared acting as a Chair for the Transforming Health and Care Systems EU funding board.

All other authors had no interests to declare.

## Appendix ASupplementary data

Appendices

## References

[1] World Health Organisation (2024). Leading health agencies outline updated terminology for pathogens that transmit through the air [Internet]. https://www.who.int/news/item/18-04-2024-leading-health-agencies-outline-updated-terminology-for-pathogens-that-transmit-through-the-air.

[2] Beggs C.B., Abid R., Motallebi F., Samad A., Venkatesan N., Avital E.J. (2024). Airborne transmission of SARS-CoV-2: the contrast between indoors and outdoors. Fluids.

[3] Mikszewski A., Stabile L., Buonanno G., Morawska L. (2022). The airborne contagiousness of respiratory viruses: a comparative analysis and implications for mitigation. Geosci Front.

[4] World Health Organisation (WHO) (2020). Coronavirus disease (COVID-19). Situation Report – 172 [Internet].

[5] Christian H. (2021). https://www.nhsconfed.org/articles/clean-air-nhs-most-powerful-weapon-against-covid-19.

[6] British Medical Association (BMA) (2023). COVID-19: impact of the pandemic on healthcare delivery [Internet].

[7] NHS England (2023).

[8] Settimo G., Gola M., Capolongo S. (2020). The relevance of indoor air quality in hospital settings: from an exclusively biological issue to a global approach in the Italian context. Atmosphere.

[9] Oberst M., Heinrich A. (2021). Effect of a mobile room air filter on the aerosol burden in surgical examination rooms against the background of the COVID-19 pandemic. Unfallchirurg.

[10] Tricco A.C., Langlois E., Straus S.E., World Health Organization (2017).

[11] Popay J., Roberts H., Sowden A. (2006).

[12] Hong Q.N., Fàbregues S., Bartlett G. (2018). The Mixed Methods Appraisal Tool (MMAT) version 2018 for information professionals and researchers. Educ Inf.

[13] Conway Morris A., Sharrocks K., Bousfield R. (2022). The removal of airborne severe acute respiratory syndrome coronavirus 2 (SARS-CoV-2) and other microbial bioaerosols by air filtration on coronavirus disease 2019 (COVID-19) surge units. Clin Infect Dis.

[14] Butler M.J., Sloof D., Peters C. (2023). Impact of supplementary air filtration on aerosols and particulate matter in a UK hospital ward: a case study. J Hosp Infect.

[15] Lu Y.R., Li Y.F., Lin M.G. (2021). Environmental monitoring and infection control of fever clinics in general hospitals during COVID-19 pandemic. Chin Sci Bull.

[16] Mousavi E.S., Pollitt K.J., Sherman J., Martinello R.A. (2020). Performance analysis of portable HEPA filters and temporary plastic anterooms on the spread of surrogate coronavirus. Build Environ.

[17] Li Y., Ching W.H., Qian H. (2007). An evaluation of the ventilation performance of new SARS isolation wards in nine hospitals in Hong Kong. Indoor Built Environ.

[18] Fennelly M., Hellebust S., Wenger J. (2023). Portable HEPA filtration successfully augments natural-ventilation-mediated airborne particle clearance in a legacy design hospital ward. J Hosp Infect.

[19] Vokurka S., Bystrická E., Svoboda T. (2014). The availability of HEPA-filtered rooms and the incidence of pneumonia in patients after haematopoietic stem cell transplantation (HSCT): results from a prospective, multicentre, eastern European study. J Clin Nurs.

[20] Buising K.L., Schofield R., Irving L. (2022). Use of portable air cleaners to reduce aerosol transmission on a hospital coronavirus disease 2019 (COVID-19) ward. Infect Control Hosp Epidemiol.

[21] Park S.Y., Yu J., Bae S. (2023). Ventilation strategies based on an aerodynamic analysis during a large-scale SARS-CoV-2 outbreak in an acute-care hospital. J Clin Virol.

[22] Ryan R.M., Wilding G.E., Wynn R.J., Welliver R.C., Holm B.A., Leach C.L. (2011). Effect of enhanced ultraviolet germicidal irradiation in the heating ventilation and air conditioning system on ventilator-associated pneumonia in a neonatal intensive care unit. J Perinatol.

[23] Salam Z.H., Karlin R.B., Ling M.L., Yang K.S. (2010). The impact of portable high-efficiency particulate air filters on the incidence of invasive aspergillosis in a large acute tertiary-care hospital. Am J Infect Control.

[24] Rezaei N., Jafari M., Nazari A. (2020). A novel methodology and new concept of SARS-CoV-2 elimination in heating and ventilating air conditioning systems using waste heat recovery. AIP Adv.

[25] Lee J.H., Rounds M., McGain F. (2022). Effectiveness of portable air filtration on reducing indoor aerosol transmission: preclinical observational trials. J Hosp Infect.

[26] Rao N.G., Kumar A., Colon C., Goswami D.Y. (2020). Impact of a new portable air purification technology device in the pediatric hospital setting–a pre-post assessment study. Cureus.

[27] Salmonsmith J., Ducci A., Balachandran R. (2023). Use of portable air purifiers to reduce aerosols in hospital settings and cut down the clinical backlog. Epidemiol Infect.

[28] Li S., Guo J., Gu Y. (2023). Assessing airborne transmission risks in COVID-19 hospitals by systematically monitoring SARS-CoV-2 in the air. Microbiol Spectr.

[29] Otter J.A., Clark L., Taylor G., Hussein A., Gargee L., Goldenberg S.D. (2023). Comparative evaluation of stand-alone HEPA-based air decontamination systems. Infect Dis Health.

[30] Gola M., Settimo G., Capolongo S. (2019). Chemical pollution in healing spaces: the decalogue of the best practices for adequate indoor air quality in inpatient rooms. Int J Environ Res Publ Health.

[31] Settimo G., Yu Y., Gola M., Buffoli M., Capolongo S. (2023). Challenges in IAQ for indoor spaces: a comparison of the reference guideline values of indoor air pollutants from the governments and international institutions. Atmosphere.

[32] Gola M., Settimo G., Capolongo S. (2020). How can design features and other factors affect the indoor air quality in inpatient rooms? Check-lists for the design phase, daily procedures and maintenance activities for reducing the air concentrations of chemical pollution. Int J Environ Res Publ Health.

[33] Seppänen O.L. (2021). Effects of Indoor Air Humidity.

[34] International Organization for Standardization (2017). High efficiency filters and filter media for removing particles from air — part 1: classification, performance, testing and marking. [Internet]. http://www.iso.org/obp/ui/#iso:std:iso:29463:-1:ed-2:v1:en.

[35] NHS England (2023). NHS Estates Technical Bulletin (NETB 2023/01A): application of HEPA filter devices for air cleaning in healthcare spaces: guidance and standards. [Internet]. http://www.england.nhs.uk/long-read/application-of-hepa-filter-devices-for-air-cleaning-in-healthcare-spaces-guidance-and-standards.

[36] Vijayan V.K., Paramesh H., Salvi S.S., Dalal A.A. (2015). Enhancing indoor air quality–the air filter advantage. Lung India.

[37] Winck J.C., Almeida S.M., Correia G., Gabriel M.F., Marques G., Silva M.G. (2022). A call for a national strategy for indoor air quality. Pulmonology.

[38] Demeersseman N., Saegeman V., Cossey V., Devriese H., Schuermans A. (2023). Shedding a light on ultraviolet-C technologies in the hospital environment. J Hosp Infect.

