# Supplementary material for: Interventions used to reduce infectious aerosol concentrations in hospitals—a review
Source: eClinicalMedicine. 2024 Dec 18;79:102990. doi: 10.1016/j.eclinm.2024.102990 (PMC11718292; doi:10.1016/j.eclinm.2024.102990)
Supplement: Appendices [file mmc1.docx]

**Appendices**

**1) Search Strategy for preliminary search – PubMed**

***Themes***

***#1 Outcome terms***

Exp Virus Diseases/ OR Exp Respiratory Tract Infections/ OR “respiratory infection*”.ti,ab. OR “respiratory virus*”.ti,ab. OR “respiratory tract infections”.ti,ab.  OR Exp Pneumonia/ OR Exp COVID-19/ OR Exp SARS-CoV-2/ OR Exp Coronavirus/ OR Coronavirus*.ti,ab. OR “Coronavirus infection*”.ti,ab. OR 2019-nCoV.ti,ab. OR 2019 ncov.ti,ab. OR nCov.ti,ab. OR Covid19.ti,ab. OR SARSCoV-2.ti,ab. OR “novel coronavirus”.ti,ab. OR “novel corona virus”.ti,ab. OR covid*.ti,ab. OR “severe acute respiratory syndrome”.ti,ab. OR “coronavirus 2”.ti,ab. OR “coronavirus disease”.ti,ab. OR “corona virus disease”.ti,ab. OR “new coronavirus”.ti,ab. OR “new corona virus”.ti,ab. OR “new coronaviruses”.ti,ab. OR “novel coronaviruses”.ti,ab. OR Sars.ti,ab. OR “sars corona virus”.ti,ab. OR “respiratory infectious disease*”.ti,ab. OR “acute respiratory disease*”.ti,ab. OR “influenza like illness”.ti,ab. OR Exp Pandemics/ OR Pandemic*.ti,ab. OR “respiratory disease”.ti,ab.

***#2 Intervention terms***

("filtrate"[All Fields] OR "filtrated"[All Fields] OR "filtrates"[All Fields] OR "filtrating"[All Fields] OR "filtration"[MeSH Terms] OR "filtration"[All Fields] OR "filtrations"[All Fields] OR ("recirculate"[All Fields] OR "recirculated"[All Fields] OR "recirculates"[All Fields] OR "recirculating"[All Fields] OR "recirculation"[All Fields] OR "recirculations"[All Fields]) OR ("ventilated"[All Fields] OR "ventilates"[All Fields] OR "ventilating"[All Fields] OR "ventilation"[MeSH Terms] OR "ventilation"[All Fields] OR "ventilate"[All Fields] OR "ventilations"[All Fields] OR "ventillation"[All Fields]) OR “natural ventilation”[All Fields] OR “heating”[All Fields] OR "HVAC"[All Fields] OR (("mechanical"[All Fields] OR "mechanically"[All Fields] OR "mechanicals"[All Fields] OR "mechanics"[MeSH Terms] OR "mechanics"[All Fields] OR "mechanic"[All Fields])

AND "systems"[All Fields]) OR "system"[All Fields] OR "system s"[All Fields] OR "systems"[All Fields])) OR "Airconditioning"[All Fields] OR (("ventilated"[All Fields] OR "ventilates"[All Fields] OR "ventilating"[All Fields] OR "ventilation"[MeSH Terms] OR "ventilation"[All Fields] OR "ventilate"[All Fields] OR "ventilations"[All Fields] OR "ventilator s"[All Fields] OR "ventilators, mechanical"[MeSH Terms] OR ("ventilators"[All Fields]

AND "mechanical"[All Fields]) OR "mechanical ventilators"[All Fields] OR "ventilator"[All Fields] OR "ventilators"[All Fields] OR "ventillation"[All Fields]) OR (("indoor"[All Fields] OR "indoors"[All Fields]) ("airflow"[All Fields] OR "airflows"[All Fields]) OR ("aerosol s"[All Fields] OR "aerosolic"[All Fields] OR “aerosol generating procedures”[All Fields] OR “aerosol generating procedure”[All Fields] OR "aerosolization"[All Fields] OR "aerosolizations"[All Fields] OR "aerosolize"[All Fields] OR "aerosolized"[All Fields] OR "aerosolizer"[All Fields] OR "aerosolizes"[All Fields] OR "aerosolizing"[All Fields] OR "aerosols"[MeSH Terms] OR "aerosols"[All Fields] OR "aerosol"[All Fields]) OR (("airborn"[All Fields] OR "airborne"[All Fields])

AND ("precaution"[All Fields] OR "precautions"[All Fields]))) AND (y_1[Filter])

***#3 Environment terms***

Hospitals [tiab] OR Clinic [tiab] OR Clinics [tiab] OR Infirmary [tiab] OR “healthcare facilities” [tiab] OR “healthcare facility” [tiab] OR “health care facilities” [tiab] OR “health care facility” [tiab] OR “medical centre” [tiab] OR “health centre” [tiab] OR “emergency department” [tiab] OR ED [tiab] OR “accident and emergency” [tiab] OR “a and e” [tiab] OR Exp hospital* OR “emergency room” [tiab] OR Ward [tiab]

***Strategy***

For the initial exploration of results to determine sensitivity versus breadth we are looking at:

1. + 2 + 3

**2) Final strategy and search for MEDLINE, Web of Science and The Cochrane Library**

***#1 Outcome terms***

“respiratory infection” OR “respiratory virus” OR “respiratory tract infections” OR Pneumonia

OR COVID-19 OR SARS-CoV-2 OR influenza

***#2 Intervention terms***

“HEPA filters” OR HEPA OR “UV-C systems” OR UV-C OR “laminar air flow systems” OR “filtration” OR “recirculation” OR “airflow”

1 + 2

Date range: No time restriction

**Website searching**

1. The Cochrane Library: 1867 results
2. Web of Science: 3344 results
3. MEDLINE: 1298 results

**3) Inclusion Criteria**

| **Inclusion** | **Exclusion** |
| --- | --- |
| ***Intervention*** |  |
| Interventions improving aerosol concentrations e.g. ventilation (natural or mechanical), recirculation, filtration | Any intervention that does not outline improving aerosol concentrations.  Face masks and respirators. |
| ***Outcome*** |  |
| Any respiratory virus(es)/infection(s)  COVID-19  Pneumonia  Influenza | Any other nonrelated health problem |
| ***Environment*** |  |
| Any hospital/healthcare setting e.g., clinics, hospitals, a&e, wards | An environment that is not in a healthcare environment which includes GP practices, care homes, clinics and dentists. |
| ***Methodology*** |  |
| RQ1: description of intervention used to improve aerosol concentrations in hospitals | All RQs:  Systematic reviews, meta-analyses, dissertation, editorials and conference abstracts  No feasibility studies |
| RQ2 &3:  Trials, experimental studies, quantitative studies (observational) |  |
| ***Other*** |  |
| Any publication date  Any language  All countries  Described/evaluated in peer-reviewed publications |  |

**4) Mixed Methods Appraisal Tool (MMAT) scoring**

| **AUTHORS** | **Quantitative non-randomized study** | | | | | |
| --- | --- | --- | --- | --- | --- | --- |
|  | Are the participants representative of the target population? | Are measurements appropriate regarding both the outcome and intervention (or exposure)? | Are there complete outcome data? | Are the confounders accounted for in the design and analysis? | During the study period, is the intervention administered (or exposure occurred) as intended? | Total score |
| Conway Morris *et al.* [13] | Yes | Yes | Yes | No | Yes | 4/5 |
| Butler *et al.* [14] | N/A | Yes | No | No | Yes | 2/4 |
| Mousavi *et al.* [16] | N/A | Yes | Yes | No | Yes | 3/4 |
| Oberst & Henrich [9] | Yes | Yes | Yes | No | Yes | 4/5 |
| Fennelly *et al.* [18] | N/A | Yes | Yes | No | Yes | 3/4 |
| Vokurka *et al.* [19] | Yes | No | Yes | No | Yes | 3/5 |
| Buising *et al.* [20] | N/A | Yes | Yes | No | Yes | 3/4 |
| Park *et al.* [21] | N/A | Yes | Yes | No | Yes | 3/4 |
| Ryan *et al.* [22] | Yes | Yes | Yes | No | Yes | 4/5 |
| Salam *et al.* [23] | Yes | Yes | Yes | Yes | Yes | 5/5 |
| Lee *et al.* [25] | N/A | Yes | Yes | No | Yes | 3/4 |
| Rao *et al.* [26] | Yes | Yes | Yes | Yes | Yes | 5/5 |
| Salmonsmith *et al.* [27] | N/A | Yes | Yes | No | Yes | 3/4 |
| Otter *et al.* [29] | N/A | Yes | Yes | No | Yes | 3/4 |
| **AUTHORS** | **Quantitative descriptive study** | | | | | |
|  | Is the sampling strategy relevant to address the research question? | Is the sampling representative of the target population? | Are the measurements appropriate? | Is the risk of nonresponse bias low? | Is the statistical analysis appropriate to answer the research question? | Total score |
| Lu *et al.* [15] | N/A | N/A | Yes | N/A | No | 1/2 |
| Li *et al.* [17] | N/A | N/A | Yes | N/A | No | 1/2 |
| Rezaei *et al.* [24] | N/A | N/A | Yes | N/A | No | 1/2 |
| Li *et al.* [28] | N/A | N/A | Yes | N/A | No | 1/2 |
